# Evaluation of potential reference genes for real time RT-PCR studies in Atlantic halibut (*Hippoglossus Hippoglossus *L.); during development, in tissues of healthy and NNV-injected fish, and in anterior kidney leucocytes

**DOI:** 10.1186/1471-2199-11-36

**Published:** 2010-05-11

**Authors:** Aina-Cathrine Øvergård, Audun Helge Nerland, Sonal Patel

**Affiliations:** 1 Institute of Marine Research, PO Box 1870 Nordnes, N-5817 Bergen, Norway; 2 The Gade Institute, University of Bergen, PO Box 7804, N-5021 Bergen, Norway

## Abstract

**Background:**

Real time RT-PCR has become an important tool for analyzing gene expression in fish. Although several housekeeping genes have been evaluated in Atlantic halibut (*Hippoglossus Hippoglossus *L.), appropriate reference genes for low copy mRNA transcripts at the earliest developmental stages have not been identified. No attempts have been reported to identify suitable reference genes in halibut infected with NNV or in stimulated halibut leucocytes. In this study, β-actin1 (ACTB1), elongation factor 1 alpha (EF1A1), hypoxanthine-guanine phosphoribosyltransferase 1 (HPRT1), ribosomal protein L7 (RPL7), tubulin beta 2C (Tubb2C), and ubiquitin-conjugating enzyme (UbcE) were evaluated as reference genes for normalization of real time RT-PCR data during Atlantic halibut development, in tissue of healthy and NNV-infected fish, and in *in vivo *and *in vitro *stimulated anterior kidney leucocytes.

**Results:**

The expression of all six genes was relatively stable from the unfertilized egg until 12 day degrees post fertilization (ddpf). However, none of the selected genes were found to be stably expressed throughout halibut development. The mRNA levels of the six genes increased from 18 ddpf, when zygotic transcription is likely to be activated, and stabilized at different time points. The Excel-based software programs BestKeeper, geNorm, and NormFinder ranked EF1A1 and UbcE as the best candidate reference genes before activation of zygotic transcription, and RPL7 and EF1A1 as the best candidates after hatching. EF1A1 and RPL7 were also listed as the best reference genes when exploring the expression levels of the six genes in various halibut organs, both in non-injected fish and in mock- and NNV-injected fish. None of the reference genes were found optimal for normalization of real time RT-PCR data from *in vitro *stimulated anterior kidney leucocytes.

**Conclusion:**

Generally, it was found that EF1A1 and RPL7 were the genes that showed least variation, with HPRT1 and UbcE as intermediate genes, and ACTB1 and Tubb2C as the least stable ones. None of the six reference genes can be recommended as reference gene candidates in ConA-PMA stimulated leucocytes. However, UbcE can be a good candidate in other experimental setups. This study emphasizes the need for reference gene evaluation, as universal reference genes have not been identified.

## Background

Real time reverse transcriptase polymerase chain reaction (real time RT-PCR) has become a widely used method for gene expression analysis, and it is a useful method for studying immune related genes and host-pathogen interactions. It is more accurate and sensitive than traditional methods like RT-PCR and northern blotting [1], but normalization of the assay is critically important as differences in loading amounts of total RNA in the RT reaction, variations in RT efficiency and RNA integrity, instrumental errors, and the presence of PCR inhibitors have to be accounted for [2]. Housekeeping genes are often used as internal reference genes. Ideally, genes chosen should have stable gene expression among individuals, organs and cells, during different developmental stages, and various experimental treatments. The housekeeping genes chosen should thus be validated for each new experimental setup. Also, the use of a single housekeeping gene has been found to be insufficient [3]. Thus, it is important to evaluate and establish a two-gene normalization strategy for normalization of real time RT-PCR data. While establishing such a strategy one should bear in mind not to use genes involved in the same biological process to avoid co-regulation.

Larvae hatching at a primitive state, followed by a long developmental period has made the farming of the marine flatfish Atlantic halibut (*Hippoglossus Hippoglossus *L.) challenging [4,5]. Several microorganisms have been associated with high mortality of halibut eggs and larvae at this stage of life when the halibut immune system is poorly developed [6]. One of the most important pathogen in economical terms affecting halibut during larval and early juvenile stages is the nervous necrosis virus (NNV). NNV is the causative agent of Viral Encephalopathy and Retinopathy (VER), and the major site for virus replication is within the central nervous system [6]. Much work has been done to characterize various NNV strains and in vaccine development [7-11]. However, analyzing halibut immune related genes in response to NNV-infection has not been optimal as suitable reference genes for such experimental setups have not been evaluated.

Several commonly used reference genes have been applied in real time RT-PCR studies of Atlantic halibut gene expression, including β-actin (ACTB), 18S rRNA, elongation factor 1 alpha (EF1A), and glyceraldehyde-3-phosphate dehydrogenase (GAPDH) [12-15]. Recently, several housekeeping genes have been evaluated during halibut development where GAPDH was found to be unsuitable as a reference gene in halibut egg and larvae [16,17]. Moreover, Fernandes *et al*. [16] found 18S rRNA to be rather stable from the two cell stage at about 1 day degree post fertilization (ddpf) to the first feeding stage. However, other genes tested, including ACTB appeared to be developmentally regulated at early stages [16], as seen in Japanese flounder (*Paralichthys olivaceus*) [18]. Also, the time points sampled before activation of zygotic transcription were not sufficient to evaluate potential reference gene candidates, and appropriate reference genes for low copy mRNA transcripts at the earliest developmental stages remains to be identified. Some potential reference genes have been analyzed in several tissues of Atlantic halibut, including ACTB and some ribosomal proteins. ACTB was found to be inappropriate for normalization of muscle samples during fasting and re-feeding of halibut males [19], but the ACTB gene expression was not tested in other tissues. Paralogous genes of ribosomal protein L19 (RPL19) and RPL22 were tested in halibut larva and in several organs of three year old halibut with promising results [20]. However, no statistical evaluation of the gene expression stability was conducted in this study, and further evaluation of ribosomal proteins as reference gene candidates is needed.

Based on earlier gene expression studies in halibut [16,17], Japanese flounder [18], medaka (*Oryzias latipes*) [21], and Atlantic salmon (*Salmo salar*) [22,23], six housekeeping genes, ACTB1, EF1A1, hypoxanthine-guanine phosphoribosyltransferase 1 (HPRT1), RPL7, tubulin beta 2C (Tubb2C), and ubiquitin-conjugating enzyme E2L3 (UbcE) were selected. In the present study, the selected genes were evaluated as reference genes for normalization of real time RT-PCR studies during halibut development from the unfertilized egg to juveniles, as well as in different organs of healthy fish. Evaluation of these genes was also carried out in tissues sampled from fish infected with NNV and in anterior kidney leucocytes isolated from *in vivo *NNV-stimulated fish and further *in vitro *stimulated with ConA-PMA. This study will give valuable information regarding reference genes for real time RT-PCR studies during halibut development, as it will give a more detailed evaluation of the earliest developmental stages before activation of zygotic transcription. Also, results may serve as a good foundation for further search of reference genes in experimental setups including infection and leucocyte stimulation, both in halibut and other fish species.

## Methods

### Fish stocks and sample collection

Halibut eggs and larvae were collected in two different experiments conducted a year apart, and reared at the Austevoll Aquaculture Reseach Station, Norway and at the Institute of Marine Research (IMR, Bergen), Norway following normal rearing conditions [4,24,25]. The larvae were fed enriched *Artemia *until weaning to commercially available dry pellets at 1150 day degrees post hatching (ddph). In the first experiment, samples were taken from unfertilized eggs at stripping, and at 0.04, 0.08, 0.17, 0.25, 0.9, 6, 12, 18, 24, 30, 36, 42, 48, and 72 ddpf, and further of larvae at 0, 18, 30, 42 and 60 ddph. In the second experiment, samples were taken from unfertilized egg at stripping, and from fertilized eggs at 0, 30, and 72 day degrees post fertilization (ddpf), and at 0, 18, 42, 60, 84, 102, 126, 144, 168, 186, 210, 228, 252, 270, 318, 354, 438, 522, 606, 690, 774, 858, 954, 1026, 1110, 1194, 1278, 1458, and 1638 ddph. For developmental stages corresponding to the different time-points, see Pittman *et al*. [26] and Patel *el al*. [27]. For total RNA purification, five eggs or larvae were pooled at each time point until 210 ddph. From 228 ddph to 1638 ddph, either one individual, or a pool of 2-5 larvae or juveniles depending on the size and biomass, were employed after dividing them in two just behind the intra-peritoneal cavity, and the anterior part was used for total RNA isolation. For each time point four parallels were purified.

Generally, fish 6 months or older were acclimatized upon arrival at IMR (Bergen), Norway, and reared in seawater (34 ppm salinity) at 9°C, and fed commercial pellets twice a day. If injected, fish were anesthetized with benzocain (The Norwegian medicine depot) at a concentration of 60 mg/l seawater, while for tissue sampling an overdose of benzocain was employed.

Four individuals, approximately 1 year old and weighing between 70 - 150 g, were obtained from Austevoll Aquaculture Reseach station, Norway. For total RNA isolation, samples were collected from thymus, spleen, skin, heart, anterior and posterior kidney, pectoral fin, gills, brain, liver, anterior and posterior gut, red and white muscle, stomach, and eye.

Fish approximately 6 months old and weighing approximately 30 g, were obtained from Aga Marine, Bømlo, Norway. They were injected *intra peritoneal *(*i.p*) with either 200 μl of L-15 medium (Sigma) or 200 μl of 1 × 10^8.5 ^TCID_50 _NNV. The NNV used was isolated from natural outbreak affecting halibut [28], and propagated in cell culture as described previously [29]. Samples from brain, retinal nerve, thymus, anterior kidney, spleen, and liver were taken from six individuals from both NNV-injected and mock-injected group (L-15 medium injected group) at 6, 12, 24, 72 hours (hrs) and 1, 2, 3, and 4 weeks post-injection. Anterior kidney leucocytes were isolated as described previously [30] from four fish from each group 10 weeks post-injection. The isolated leucocytes were further divided into two aliquots and plated out in 24-well culture plates. One aliquot served as a non-stimulated control group and the other group was stimulated with 10 μg/ml with 5 ng/ml phorbol myristate acetate (Calbiochem) (ConA-PMA) as previously described [30]. Cells were harvested at 0 hrs (non-stimulated) and from both non-stimulated control and ConA-PMA stimulated group after 4, 12, 18, and 24 hrs of incubation, pelleted, and frozen at -80°C until use.

All eggs, larva, and tissue samples were snap-frozen in liquid nitrogen immediately after sampling, and stored at -80°C until use. All fish experiments were performed according to national legislation and approved by the Norwegian animal experimentation board.

### Isolation of total RNA and cDNA synthesis

Total RNA from developmental stages and from healthy fish was isolated using TRI reagent (Sigma) according to the Trizol reagent protocol described by Invitrogen, with a few modifications as described previously [14]. The tissue samples from injected fish were purified using the iPrep™ TRIzol^® ^Plus RNA Kit (Invitrogen). Total RNA from leucocytes was purified by a combined Trizol (Invitrogen) and RNeasy (Qiagen) method. Briefly, the aqueous phase from the chloroform phase separation (Trizol method) were added 1 volume of 70% ethanol, transferred to an RNeasy spin column, and total RNA was further purified according to the RNeasy^® ^Mini Handbook (Qiagen).

The concentration and the purity of the total RNA were assessed with a NanoDrop Spectrophotometer (NanoDrop Technologies), and the quality of random samples was analyzed with an Agilent 2100 Bioanalyzer (Agilent Technologies). A 260/280 nm absorbance ratio above 1.8 was accepted as pure, and RNA integrity numbers (RIN) above 5 were considered as good quality total RNA. Total RNA was reversed transcribed using a Reverse Transcriptase Core Kit (Eurogentec) and random nonamers as primers. All total RNA was reversed transcribed in 30 μl leucocytes where 300 ng of total RNA was used due to low total RNA concentrations. cDNA was stored at -20°C until use.

### Cloning and DNA sequencing

Expressed sequence tags (EST) representing EF1A1 [GeneBank: GE628160], ACTB1 [GeneBank: GE628929, GE629238, GE629769, GE631177, GE631405, GE631443], Tubb2C [GeneBank: GE630028, GE631294, GE631482], and HPRT1 [GeneBank: GE627320, GE631255] were identified in a sequence database generated on the basis of Atlantic halibut cDNA libraries [30].The RPL7 EST [GeneBank: EB035355] and the UbcE EST [GeneBank: CF931657] were retrieved from NCBI http://www.ncbi.nlm.nih.gov/sites/entrez[31,32]. The EF1A1 clone was amplified in XL 10-Gold^® ^ultracompetent *E. coli*, and plasmid DNA was purified using the QIAprep^® ^Spin Miniprep Kit for Microcentrifuge (Qiagen). The plasmid insert was sequenced by primer walking using BigDye^® ^Terminator v3.1 Cycle Sequencing kit and run on an ABI prism 7700 automated sequencing apparatus (Applied Biosystems). The PCR cycling was carried out as follows: 96°C for 5 min followed by 25 cycles of 96°C for 10 sec, 50°C for 5 sec, and 60°C for 4 min. The full length sequence [GeneBank: EU561357] was retrieved by RLM-RACE (Invitrogen) as described previously [14], and the PCR products were purified using QIAquick PCR purification kit (Qiagen) and sequenced as described above.

To confirm the EST sequences of the ACTB1, HPRT1, RPL7, Tubb2C, and UbcE gene, primers were designed based on EST alignments. Amplification of cDNA was performed in 25 μl reactions GoTaq^® ^Flexi DNA Polymerase with (Promega), using 200 nM of each PCR primers, and 0.5 μl cDNA. The PCR cycling was carried out as follows: 95°C for 5 min followed by 30 cycles of 95°C for 30 s, 55°C for 30 s, 72°C for 1 min per kb, and finally 72°C for 7 min. The PCR products were enzymatically cleaned using Shrimp Alkaline Phosphatase in combination with Exonuclease I (USB Corporation), and sequenced as described above. To confirm the exon-exon boundaries, genomic sequences [ACTB1; GeneBank: GQ465758, EF1A1; GeneBank: EU561358, HPRT1; GeneBank: GQ465759, RPL7; GeneBank: FJ008911, Tubb2C; GeneBank: GQ465760, and Ubc; GeneBank: FJ008912] were amplified by PCR as described above, using 100 ng of halibut genomic DNA purified from whole blood erythrocytes by the phenol/chloroform method, protocol I for frozen blood [33] as template. The PCR products were cloned using the TOPO TA Cloning^® ^Kit for Sequencing (Invitrogen). The plasmids were purified and the inserts were sequenced as described above.

### Primer and probe design

Primers and probes were designed with Primer express software 3.0 (Applied Biosystems), according to the manufacturer's guidelines. Either the probe or one of the primers was designed such that they spanned an exon-exon boundary, to avoid amplification of genomic DNA. Both primers and probes were screened for homo- or cross-dimers and hairpin structures that could affect the efficiency of the PCR reaction. Five-point standard curves of 4-fold dilution series (1:1 - 1:256) from pooled cDNA were used for calculation of the PCR efficiency, given by the equation E% = (10^1/slope ^- 1) × 100 [34], and for revealing PCR poisoning. The slope was calculated from the linear regression model fitted from the log-transformed cDNA concentrations plotted against the Ct values. Generally, a PCR efficiency between 90-110% is considered acceptable (Applied Biosystems). The primer and probe sequences with corresponding PCR efficiencies are listed in Table 1. Retinal nerve samples were analyzed for the presence of viral RNA using a real time RT-PCR assay previously described [35].

**Table 1:** Primers and probes used for real time RT-PCR analysis.

Gene	Forward	Revers	Probe	E%
ACTB1	CGGTCGTACCACAGGTATCGT	GGATCTTCATCAGGTAGTCAGTCAGA	TCTGGTGACGGT-MGB	90
EF1A1	CCATGGTTGTGGAGTCCTTCTC	GATGACACCGACAGCCACTGT	CTCCCCTCGGTCGTTTCGCTGTG	96
HPRT1	GTGGACTTCATTCGCCTCAAG	TCTCCACCGATAACTTTGATTTCA	ACTGGTCGTTACAGTAGC-MGB	98
RPL7	GAAGGCTCTCGGCAAATATGG	GCCAACTGTGTAAATCTCATGGAT	TCCTCGACGCAGATGA-MGB	94
Tubb2C	GCTGGACAGGATAAACGTGTATTTACA	GTGCCCGGCTCCAGATC	CCTCAGGCGGTAAAT-MGB	93
UbcE	GAACTGGAAACCAGCGACAAA	CGCTCACCAGAGCAATGAGA	ACCATCCAAGTGATCAATA-MGB	98

The primers and probes are listed 5' → 3' direction with the corresponding PCR efficiency (E%). All probes are marked with 6-carboxyfluorescein (6FAM). Abbreviation: MGB - minor groove binder.

### Real time RT-PCR assay and data analysis

The PCR reaction mix contained 1× TaqMan Fast PCR Master Mix (Applied Biosystems), 900 nM of each primer, 200 nM TaqMan probe The PCR cycling was carried out as follows: 95°C for 20 sec, 40 cycles of 95°C for 1 sec followed by 60°C for 20 sec. Two technical replicates were run for each sample on the 7900 HT Fast Real-Time PCR System (Applied Biosystems), and if the percent deviation was below 5% the mean Ct values for each sample were used for further analysis. Non template controls and samples negative for the reverse transcriptase enzyme were included. All assays were tested for amplification of genomic DNA, using 200 ng of genomic DNA as template in the real time RT-PCR reaction. The data collected was analyzed by the Excel-based programs BestKeeper [36], geNorm [3], and NormFinder [37]. Three programs were used as none of these are accepted methods on their own for evaluation and ranking of reference genes, thus by using three independent methods a more reliable result was expected.

## Results and Discussion

For real time RT-PCR analysis, it is essential to use good quality total RNA with high degree of purity [38]. High concentrations of contaminants may give PCR poisoning and decrease reverse transcriptase activity, while degraded total RNA can give inaccurate measurement of target gene RNA if the region of interest is degraded before analysis. In the present study, RNA quality and purity measured by Bioanalyzer and NanoDrop respectively, showed that high quality total RNA was extracted with RIN values above 6, normally close to 10, and ratios of 260/280 nm absorbance between 1.8 and 2.1. When using the 2^-ΔΔCt ^method it is crucial to have a primer and probe design that gives PCR efficiency close to 100% when the correlation coefficient (R^2^) of the linear regression line is close to one [39]. The PCR efficiency of the six assays established in this study was between 90 and 98% (Table 1), and PCR poisoning could not be seen (R^2 ^close to 1). No signal was detected when samples lacking the reverse transcriptase enzyme or genomic DNA were analyzed, indicating that the assays did not detect any genomic DNA contaminants.

When the collected data was analyzed by BestKeeper, geNorm, and NormFinder, often the three software programs deviated in the ranking order of the analyzed genes reflecting the differences in the estimation approach by the three programs. Both BestKeeper and geNorm uses a pair-wise comparison approach, and are highly dependent on the assumption that none of the genes being analyzed are co-regulated [3,36]. BestKeeper ranks reference genes according to a correlation with a BestKeeper index, calculated based on the geometric mean of the Ct values of the candidate reference genes with a standard deviation (SD) below 1. The geNorm program calculates an *M *value that corresponds to the average pair-wise variation of a single reference gene to all other genes, and allows for a repetitive procedure where the least stably expressed candidate is removed and new *M *values are calculated. The gene with the lowest *M *value should be the most stably expressed gene, and the recalculation of the *M *values can alter the ranking of the reference genes and decrease the *M *values (Table 2). NormFinder on the other hand uses a model-based approach and ranks according to a minimal combined inter- and intra-group expression variation [37], and should be a more robust approach. However, in all the three programs used it seemed like the ranking of a given reference gene was highly dependent on the set of candidate reference genes included in the analysis. Not only was the reliability dependent on the assumption of minimal co-regulation, but also that the set of reference genes included in the analysis were relatively stably expressed. In the present study, the chosen reference genes were highly deviating in the expression stability. Often, two of the selected genes had a high degree of variation within the given sample set, one or two of the genes were intermediate, and two or three genes were relatively stable in expression. Seemingly, the software programs often chose the intermediate reference gene as the best candidate, as the expression pattern of this gene was more similar to the general expression pattern than to the most stably expressed gene. Therefore, conclusions drawn from this study were not based on the ranking order made by the three software programs alone.

**Table 2:** Average expression stability (*M *values) of the six potential reference genes according to geNorm.

Experiment	Organ	Treatment	ACTB1	EF1A1	HPRT	RPL7	Tubb2C	UbcE
Development	Larva	Dev1	0.365	0.363 (0.216)	0.389	0.391 (0.279)	0.434	0.360 (0.247)
		Dev2	0.885	0.529 (0.393)	0.583 (0.435)	0.483 (0.327)	0.592	0.529
Adult	All	None	1.464	0.782 (0.456)	0.865 (0.557)	0.774 (0.461)	1.025	0.797
Infection	All	Mock	2.008	0.969 (0.502)	1.196 (0.656)	1.011 (0.503)	1.492	1.072
		NNV	1.985	0.953 (0.458)	1.167 (0.640)	0.972 (0.477)	1.460	1.054
	Brain	Mock	0,620	0,363	0,330 (0,233)	0,422	0,352 (0,248)	0,314 (0,227)
		NNV	1,088	0,508 (0,332)	0,500 (0,288)	0,549	0,693	0,496 (0,291)
	Eye	Mock	0,916	0,500 (0,354)	0,483 (0,393)	0,532 (0,362)	0,606	0,490
		NNV	0,864	0,451 (0,351)	0,409 (0,258)	0,462	0,527	0,414 (0,278)
	Thymus	Mock	0,570	0,342 (0,271)	0,321 (0,256)	0,387	0,369	0,321 (0,254)
		NNV	0,791	0,429 (0,305)	0,409	0,440	0,420 (0,270)	0,394 (0,267)
	Spleen	Mock	0,750	0,524 (0,400)	0,577 (0,445)	0,526 (0,388)	0,567	0,514
		NNV	1,359	0,632 (0,367)	0,701	0,682 (0,410)	0,988	0,656 (0,461)
	Ant. kidney	Mock	0,750	0,478 (0,326)	0,618	0,507 (0,386)	0,564	0,455 (0,381)
		NNV	0,668	0,441 (0,314)	0,569	0,442 (0,341)	0,486	0,428 (0,370)
	Liver	Mock	1,080	0,739 (0,429)	0,793 (0,510)	0,678 (0,407)	1,091	0,664
		NNV	0,968	0,623 (0,311)	0,697 (0,408)	0,601 (0,326)	0,939	0,606
Leucocytes	A. kidney	All	0.738	0.732 (0.321)	0.719	0.627 (0.335)	0.838	0.676 (0.304)

Low *M *values indicate high expression stability, and recalculated *M *values are given in brackets. The recalculated *M *values are given for the three most stably expressed genes according to geNorm, after elimination of the three less stable genes.

Abbreviations: Dev 1 - developmental stages before activation of zygotic transcription, Dev 2 - developmental stages after hatching, A. kidney - anterior kidney, Mock - mock-injected, NNV - NNV-injected.

### Expression of candidate reference genes during halibut development

**Figure 1:**
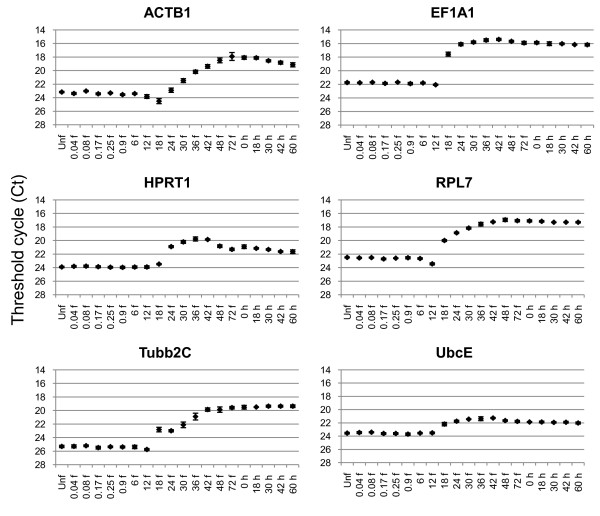
**Experiment 1**. Expression profiles of the six potential reference genes during Atlantic halibut early development. Data is shown from unfertilized egg until 60 ddph as average Ct values ± SD where n = 4. Abbreviations: Unf - unfertilized, f - day degrees post fertilization, h - day degrees post hatching.

As there is a delay between fertilization and the activation of zygotic transcription, many gene products are deposited in the egg during oogenesis to execute certain basic cellular functions [40]. The development of Atlantic halibut eggs and larvae progresses through major changes throughout development [26], evidently the activation of zygotic transcription is a major factor affecting the expression of several halibut housekeeping genes [16]. All six reference genes analyzed here were detected likely as maternal transcripts in the halibut eggs, as seen in other studies of gene expression during early teleost development [16,18,41-43]. The two sampling series, which were taken one year apart and with different brood-stock, revealed concurrence in developmental regulation of the genes with stage-specific expression stability, and the halibut eggs showed a relatively high abundance in mRNA storage (Table 3). All genes were found to have a relatively stable expression level from the unfertilized egg until 12 ddpf (Figure 1). After 12 ddpf the mRNA level of all genes except ACTB1 increased, as the zygotic transcription was likely to be activated, and stabilized at different time points. A minor drop in the ACTB1 mRNA level at 18 ddpf could be seen, and an increase in the mRNA level was not present until 24 ddpf. ACTB1 expression was previously reported during early halibut development [16], correlating with the expression pattern reported here. However, a more accurate estimate of the onset of zygotic transcription was shown in the present study.

**Table 3:** Average Ct values ± SD of the six reference genes in the different experimental setups.

Experiment	Sample	Treatment	ACTB1	EF1A1	HPRT	RPL7	Tubb2C	UbcE
Development	Larva	Dev1	23.7 ± 0.6	21.9 ± 0.3	24.2 ± 0.7	22.8 ± 0.4	25.8 ± 0.8	23.7 ± 0.3
		Dev2	21.3 ± 1.5	16.5 ± 0.7	22.3 ± 0.8	17.2 ± 0.6	20.5 ± 0.9	22.9 ± 0.8
Adult	All	None	21.2 ± 1.7	17.0 ± 0.5	22.3 ± 0.7	19.2 ± 0.6	20.7 ± 1.1	23.4 ± 0.8
Infection	All	Mock	21.0 ± 2.5	16.5 ± 0.7	21.4 ± 0.8	17.5 ± 0.8	19.9 ± 1.3	22.2 ± 0.9
		NNV	21.0 ± 2.5	16.5 ± 0.8	21.4 ± 0.8	17.4 ± 0.8	19.8 ± 1.2	22.1 ± 0.9
	Brain	Mock	24.1 ± 0.7	17.2 ± 0.3	21.5 ± 0.3	18.4 ± 0.4	18.5 ± 0.3	22.2 ± 0.3
		NNV	23.9 ± 1.2	17.3 ± 0.5	21.5 ± 0.4	18.3 ± 0.5	18.6 ± 0.6	22.1 ± 0.4
	Eye	Mock	22.6 ± 1.0	17.3 ± 0.4	22.8 ± 0.4	18.3 ± 0.4	19.8 ± 0.4	22.3 ± 0.3
		NNV	22.7 ± 0.9	17.3 ± 0.5	22.8 ± 0.4	18.3 ± 0.4	19.9 ± 0.5	22.3 ± 0.4
	Thymus	Mock	19.0 ± 0.6	16.0 ± 0.3	20.9 ± 0.3	16.9 ± 0.3	19.0 ± 0.4	21.4 ± 0.3
		NNV	19.1 ± 1.0	16.1 ± 0.5	20.9 ± 0.6	16.9 ± 0.5	19.0 ± 0.6	21.4 ± 0.5
	Spleen	Mock	18.7 ± 1.0	16.0 ± 0.5	21.3 ± 0.6	16.9 ± 0.4	20.5 ± 0.8	21.8 ± 0.7
		NNV	18.9 ± 1.6	16.0 ± 0.5	21.2 ± 0.7	16.9 ± 0.7	20.2 ± 0.9	21.6 ± 0.7
	A. kidney	Mock	18.6 ± 1.1	15.8 ± 0.5	21.1 ± 0.4	16.7 ± 0.4	19.7 ± 0.8	21.8 ± 0.7
		NNV	18.5 ± 0.7	15.8 ± 0.4	21.1 ± 0.6	16.7 ± 0.6	19.6 ± 0.6	21.6 ± 0.5
	Liver	Mock	23.2 ± 1.3	16.8 ± 0.5	20.9 ± 0.5	17.5 ± 0.5	21.7 ± 1.2	23.7 ± 0.7
		NNV	23.0 ± 1.2	16.6 ± 0.4	20.8 ± 0.4	17.5 ± 0.5	21.6 ± 1.1	23.5 ± 0.7
Leucocytes	A. kidney	Mock	24.4 ± 1.1	18.9 ± 1.0	27.8 ± 0.8	19.9 ± 0.8	24.5 ± 0.8	24.0 ± 0.9
		NNV	24.1 ± 1.0	18.6 ± 0.7	27.0 ± 1.1	19.5 ± 0.6	23.8 ± 1.2	23.6 ± 0.7
		Control	24.2 ± 1.1	18.4 ± 0.6	27.1 ± 1.1	19.4 ± 0.6	23.8 ± 1.1	23.3 ± 0.5
		ConAPMA	24.4 ± 1.1	19.1 ± 0.9	27.8 ± 1.0	20.0 ± 0.7	24.4 ± 1.0	24.2 ± 0.7

Abbreviations: Dev 1 - developmental stages before activation of zygotic transcription, Dev 2 - developmental stages after hatching, A. kidney - anterior kidney, Mock - mock-injected, NNV - NNV-injected, Control - non-stimulated control cells, ConA-PMA - cells *in vitro *stimulated with ConA-PMA.

**Figure 2:**
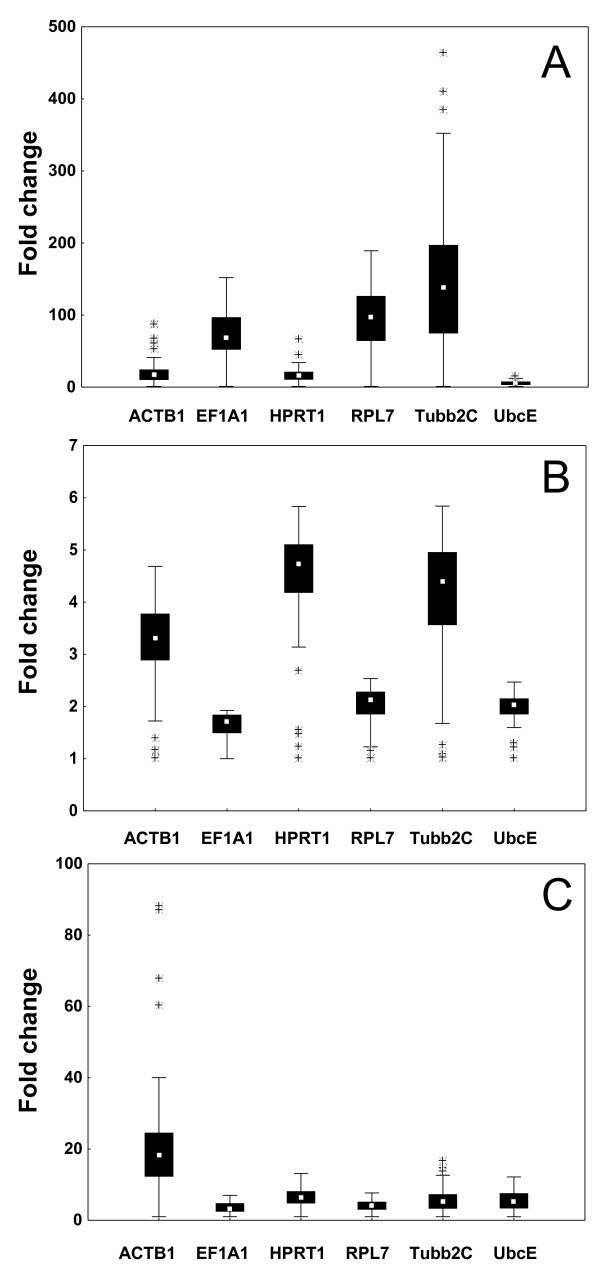
**Experiment 2**. Variation of the potential reference genes during Atlantic halibut development presented as fold change for A) developmental stages from the unfertilized egg until 1638 ddpf, B) developmental stages from the unfertilized egg until 12 ddph, and C) developmental stages from hatching (0 ddph) until 1638 ddph. Expression levels of selected genes are shown as medians (square), 25th percentile to 75th percentile (boxes), non-outlier ranges (whiskers), and outliers (asterisks).

Likely, the stage specific gene expression of the selected genes would result in an invalid data analysis using BestKeeper, geNorm, and NormFinder, as the three software programs assume that the candidate reference genes chosen are not co-regulated [3,36,37]. The data collected throughout the developmental stages was therefore not analyzed by the three software programs. However, UbcE showed the smallest level of regulation during halibut development (Figure 2A), as it decreased from an average Ct value of 23.7 before 12 ddpf to 22.9 after hatching (Table 3). This is supported by findings in Japanese flounder where UbcE, during embryogenesis, was found to be the second best reference gene after 18S rRNA [18]. Two studies have found 18S rRNA to be rather stable during Atlantic halibut development with no developmental regulation [16,17]. But due to the lack of introns [44], high expression level [45], and as the mRNA fraction of total RNA is not always represented properly by the rRNAs expression level [46], 18S rRNA is not an optimal candidate for normalization of low copy mRNA target genes. However, it can be questioned whether the use of maternal and zygotic transcripts as references within the same experiment would be optimal, and thus one should be critical in the use of a reference gene throughout development.

All genes were relatively stable from the unfertilized egg until 12 ddpf, although, EF1A1, UbcE, and RPL7 showed least variation in expression (Figure 2B). After hatching, all genes appeared to be stabilized at different stability levels (Figure 2C). To assess the most stably expressed candidate reference gene at the different expression levels before and after zygotic activation, the data collected was analyzed by BestKeeper, geNorm, and NormFinder. Before zygotic activation, from the unfertilized egg until 12 ddpf, EF1A1 was found to be the best candidate by all programs (Table 4). UbcE was ranked as the second best by geNorm and BestKeeper, while NormFinder identified Tubb2C as the second best gene. However, the UbcE and RPL7 expression were apparently more stable than the Tubb2C expression (Figure 2B), and the NormFinder ranking could thus be questioned. Also, BestKeeper showed a relatively good correlation between EF1A1 and UbcE (*r *= 0,789), indicating that they could be suitable for a two-gene normalization strategy. After hatching, all programs ranked RPL7 as the best gene, while Tubb2C and ACTB1 were identified as the most unstably expressed genes. The software programs differed in the ranking of the intermediate reference genes after hatching, as NormFinder listed UbcE > EF1A1 > HPRT1, geNorm listed EF1A1 > HPRT1 > UbcE, while BestKeeper listed EF1A1 > UbcE > HPRT1. The expression level of EF1A1 was seemingly more stable than UbcE (Figure 2C), and thus RPL7 and EF1A1 could be recommended for normalization after hatching, consistent with previous reports [17,21]. However, they cannot be recommended for a two-gene normalization strategy, as both genes are involved in protein biosynthesis, and some co-regulation may be expected. If a two-way normalization strategy is to be chosen, UbcE in combination with RPL7 or EF1A1 is likely to be the best alternative amongst the genes analyzed, as BestKeeper estimated a higher inter-gene correlation for RPL7/UbcE (*r *= 0.829) and EF1A1/UbcE (*r *= 0,769) than for RPL7/HPRT1 (*r *= 0.828) and EF1A1/HPRT1 (*r *= 0.666).

**Table 4:** Ranking output of the six reference genes according to their expression stability.

Experiment	Organ	Treatment	ACTB1			EF1A1			HPRT			RPL7			Tubb2C			UbcE		
			**N**	**G**	**B**	**N**	**G**	**B**	**N**	**G**	**B**	**N**	**G**	**B**	**N**	**G**	**B**	**N**	**G**	**B**
Development	Larva	Dev1	5	4	5	1	1	1	4	5	3	6	3	6	2	6	4	3	2	2
		Dev2	6	6	6	3	2	2	4	3	4	1	1	1	5	5	5	2	4	3
Adult	All	None	6	6	6	3	1	1	4	3	3	2	2	2	5	5	5	1	4	4
Infection	All	Mock	6	6	6	1	1	1	4	3	3	3	2	2	5	5	5	2	4	4
		NNV	6	6	6	1	1	1	4	3	3	2	2	2	5	5	5	3	4	4
	Brain	Mock	6	6	5	5	4	2	2	2	3	4	5	6	3	3	4	1	1	1
		NNV	6	6	6	3	3	3	2	1	2	4	4	5	5	5	4	1	2	1
	Eye	Mock	6	6	6	3	1	1	1	3	2	4	2	3	5	5	5	2	4	4
		NNV	6	6	6	3	3	1	1	1	2	4	4	4	5	5	5	2	2	3
	Thymus	Mock	6	6	5	3	3	4	1	2	1	5	4	6	4	5	3	2	1	2
		NNV	6	6	6	5	3	4	1	4	1	4	5	5	3	2	3	2	1	2
	Spleen	Mock	6	6	6	4	2	3	5	3	4	2	1	5	3	5	2	1	4	1
		NNV	6	6	6	2	1	3	4	4	2	3	2	4	5	5	5	1	3	1
	Ant. kidney	Mock	6	6	6	3	1	3	5	4	5	2	3	4	4	5	2	1	2	1
		NNV	6	6	6	2	1	4	5	5	5	4	2	1	3	4	3	1	3	2
	Liver	Mock	6	6	6	3	2	2	4	3	4	2	1	1	5	5	5	1	4	3
		NNV	6	6	6	3	2	2	4	3	4	2	1	1	5	5	5	1	4	3
Leucocytes	A. kidney	All	4	4	5	5	2	1	2	5	4	1	3	3	6	6	6	3	1	2

The rankings are calculated by NormFinder (N), geNorm (G), BestKeeper (B). A ranking of 1 is considered the best candidate, while 6 is listed as the worst. Abbreviations: Dev1 - developmental stages before activation of zygotic transcription, Dev2 - developmental stages after hatching, A. kidney - anterior kidney, Mock - mock-injected, NNV - NNV-injected.

### Expression of candidate reference genes in organs of one year old halibut

**Figure 3:**
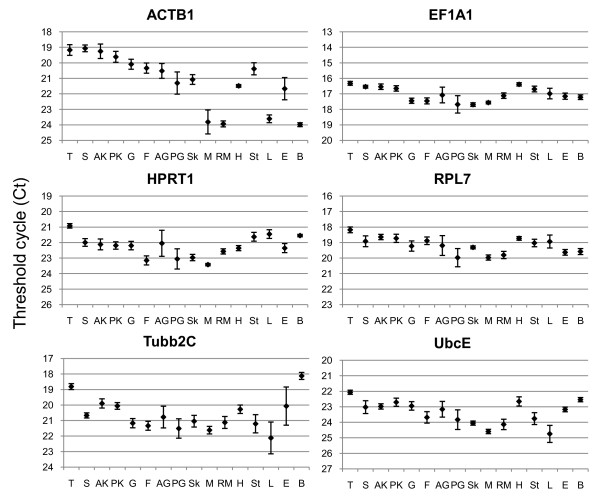
**Expression profiles of the six potential reference genes in various organs of adult Atlantic halibut**. Data is shown as average Ct values ± SD (n = 4). Abbreviations: T - thymus, S - spleen, AK - anterior kidney, PK - posterior kidney, G - gills, F - pectoral fin, AG - anterior gut, PG - posterior gut, Sk - skin, M - white muscle, RM - red muscle, H - heart, St - stomach, L - liver, E - eye, and B - brain.

When the expression levels of the six candidate reference genes were explored in various organs of one year old halibut (Figure 3), the highest mRNA level was seen for EF1A1, followed by RPL7, Tubb2C, ACTB1, HPRT1, and UbcE (Table 3). This is in accordance with the mRNA levels of ACTB1, EF1A1, HPRT1, Tubb2C, and UbcE seen during developmental stages after activation of zygotic transcription. However, the average mRNA level of RPL7 in 1 year old halibut increased by 2 Ct, indicating that RPL7 has an decrease in gene expression level at some stage after the age of 6 months. Highest variation in Ct values amongst various organs was seen for ACTB1, while EF1A1 showed least variation (Figure 3). Considering individual variations seen within organs, it could be noted that the variation was generally high in anterior and posterior gut for all genes, and also in muscle and eye for ACTB1 expression, and in stomach, liver, and eye for Tubb2C expression. However, it should be mentioned that the conclusion drawn is based on four individuals only.

Tubb2C and ACTB1 were listed as the most unstably expressed genes by all three software programs when the real time RT-PCR data from various organs was analyzed with geNorm, BestKeeper, and NormFinder. EF1A1 was ranked as the best candidate by geNorm and BestKeeper, followed by RPL7, HPRT1, and UbcE, while NormFinder proposed UbcE as the best candidate gene, followed by RPL7, EF1A1, and HPRT1 (Table 4). The different expression pattern of the six genes chosen are likely to affect the NormFinder ranking, as UbcE is seemingly not the most stably expressed gene (Figure 3). EF1A1 and RPL7 can thereby be recommended as the best candidate reference genes in one year old halibut, supported by findings in medaka [21], Atlantic salmon [23,47], and zebrafish (*Danio reio*) [43]. As mentioned previously, EF1A1 and RPL7 may be co-regulated, and HPRT1 in combination with EF1A1 (*r *= 0.656) or RPL7 (*r *= 0.633) was estimated by the BestKeeper program to be the best choice for a two-way normalization strategy in organs of adult halibut.

### Expression of candidate reference genes in organs of NNV-injected fish

**Figure 4:**
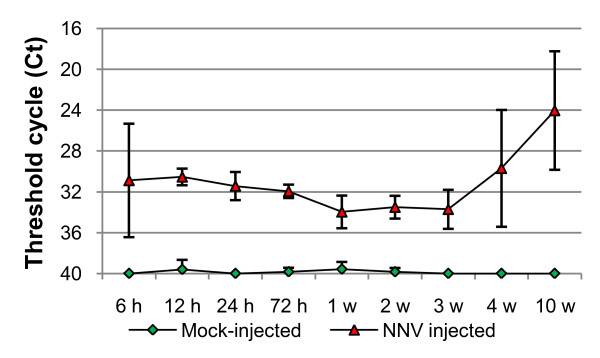
**Detection of NNV in halibut retinal nerve samples**. The data is represented as average Ct values ± SD (n = 6). Control samples that were undetected for NNV were set to 40 Ct. Abbreviations: h - hrs, w - weeks.

Generally, altered mRNA levels of host genes can be expected following viral replication and production of virus antigens, and the importance of testing reference genes under such conditions is high. A previously established assay detecting NNV RNA2 was applied to retinal nerve samples, as to ensure viral replication in the NNV-injected fish. All mock-injected fish were negative for NNV, while 99% of the injected fish were positive (Figure 4). As early as 6 hrs post-injection NNV could be detected in the retinal nerve samples, likely due to circulating virus particles. Four weeks post NNV-injection the Ct values were increased, presumably as the virus had started to propagate in the fish retina.

**Figure 5:**
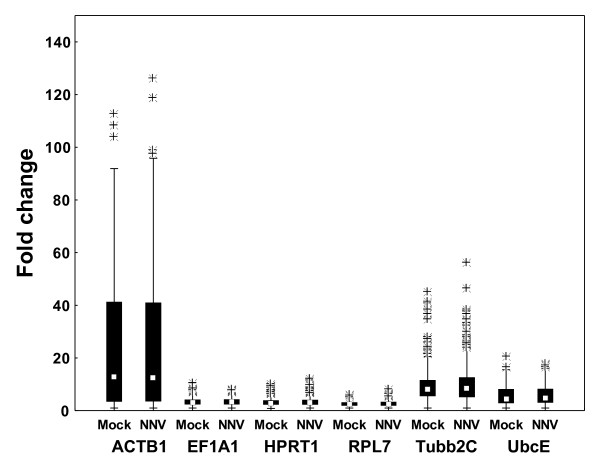
**Variation of the potential reference genes during a NNV-infection**. Data is shown as fold change of the six reference genes in all organs of mock- and NNV-injected fish. Fold change is shown as medians (square), 25th percentile to 75th percentile (boxes), non-outlier ranges (whiskers), and outliers (asterisks).

When the mRNA level of the six candidate reference genes was explored in various organs of mock-injected and NNV-injected fish, minor changes in the average Ct values (Table 3) and the general expression levels (Figure 5) were found between the two groups, presumably as the viral replication at these time points was not sufficient to reduce the level of housekeeping gene mRNA. Tubb2C was the gene showing the highest average increase in mRNA level in the NNV-infected group, with a decrease of 0.3 Ct in spleen. As expected, the highest mRNA level was seen for EF1A1, followed by RPL7, Tubb2C, ACTB1, HPRT1, and UbcE mRNA, respectively (Table 3). ACTB1 was the gene showing the highest degree of variation between organs with a SD of 2.5, with a difference in average Ct of 5.5 between anterior kidney and brain. The average expression level of RPL7 was at the same level as larvae and juveniles, increased compared to one year old fish, also when comparing the specific organs. As the fish used in the infection experiment were approximately 6 months, this supports the proposed decrease of expression between 6 months and one year. The use of RPL7 as a reference gene in this period should therefore be closely monitored, as to reveal a possible decrease in mRNA level during this developmental period.

NormFinder, geNorm, and BestKeeper showed some concurrence in the ranking of reference genes when analyzing the inter-organ variation, listing EF1A1 as the best reference gene in contrast to data published on European seabass (*Dicentrarchus labrax*) [42], and Tubb2C and ACTB1 as the worst (Table 4). However, some disagreement in the ranking of the intermediate genes HPRT1, RPL7, and UbcE was seen, as NormFinder ranked UbcE > RPL7 > HPRT1 in mock-injected group and RPL7 > UbcE > HPRT1 in NNV-injected group, while geNorm and BestKeeper ranked RPL7 > HPRT1 > UbcE in both groups. However, HPRT1 and RPL7 were seemingly lesser regulated than UbcE (Figure 5), indicating that the BestKeeper and geNorm ranking could be more reliable.

**Figure 6:**
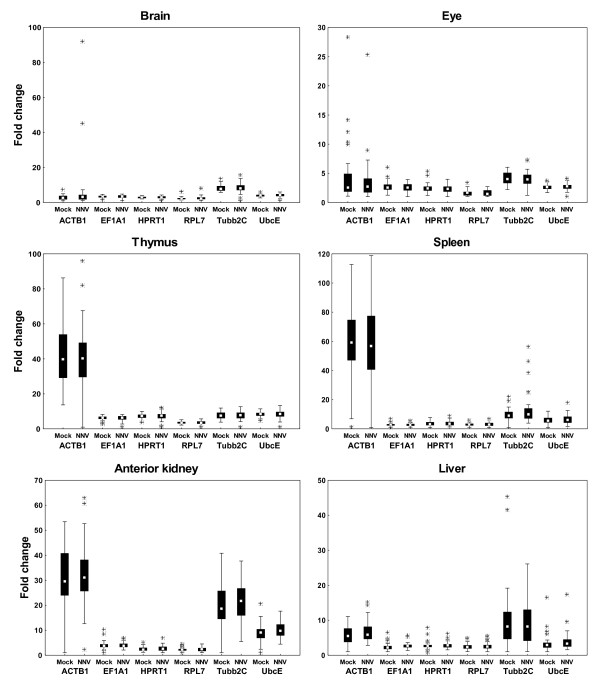
**Variation of the potential reference genes in different organs of NNV-injected fish**. Data is shown as fold change of the six reference genes in the different organs of mock- and NNV-injected fish. Fold change is shown as medians (square), 25th percentile to 75th percentile (boxes), non-outlier ranges (whiskers), and outliers (asterisks).

When analyzing the intra-organ variation the ranking of the reference genes was generally quite stable between the treatments, though with variable ranking patterns given by the different software programs (Table 4). Again, this reflects the differences in the software programs used, and the varying expression pattern between the six genes selected. As seen in tissue samples of healthy fish, ACTB1 and Tubb2C were ranked as the most unstable genes in the different tissues tested after mock- and NNV-injection, with some exceptions. The generally high variation in the ACTB1 and Tubb2C expression compared to the other genes tested, gave a basis of comparison that was highly variable between the samples. The BestKeeper software allowed for the removal of genes with a SD above 1 [36]. However, liver was the only organ where the two most unstably expressed genes, ACTB1 and Tubb2C, could be removed. The BestKeeper ranking in liver was thereby the most reasonable compared to the general expression pattern of the six genes (Figure 6), ranking RPL7 as the best gene, followed by EF1A1, UbcE, HPRT1, Tubb2C, and ACTB1 as the most unstable gene. Also, NormFinder seemingly failed to identify the most stably expressed reference gene, and listed the more intermediate gene as the best candidate instead. This gave UbcE the best ranking in brain, spleen, anterior kidney, and liver, while HPRT1 was listed as the best candidate in eye and thymus. The geNorm ranking was found to be the most reasonable compared to the general expression pattern of the reference genes (Figure 6), as the program compare the expression ratio of different genes by eliminating the worst ranked reference gene in repetitive steps. However, some apparently poor ranking was seen, illustrated by the ranking of UbcE as the best candidate reference gene in thymus, when RPL7 was seemingly the most stably expressed gene (Figure 6).

### Expression of candidate reference genes in *in vivo *and *in vitro *stimulated leucocytes

**Figure 7:**
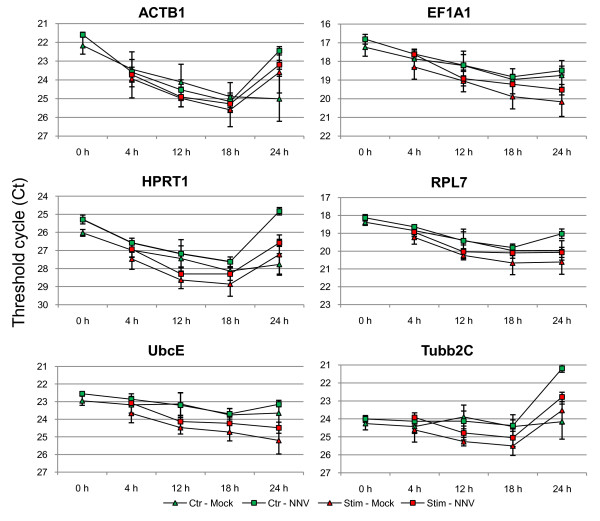
**Expression profiles of the six potential reference genes in anterior kidney leucocytes after *in vivo *stimulation with NNV and *in vitro *stimulation with ConA-PMA**. Data is shown as average Ct values ± SD (n = 4). Abbreviations: Ctr-Mock - non-stimulated control cells isolated from mock-injected fish, Ctr-NNV - non-stimulated control cells isolated from NNV-injected fish, Stim-Mock - ConA-PMA stimulated cells isolated from mock-injected fish, Stim-NNV - ConA-PMA stimulated cells isolated from NNV-injected fish.

Anterior kidney leucocytes were isolated from both mock-injected group and NNV-injected group 10 weeks post-injection and further *in vitro *stimulated with ConA-PMA, mitogens that have previously shown to stimulate fish leucocytes [48,49]. The mRNA levels of the six genes were all decreased compared to those in tissue samples (Table 3), likely due to the lesser amount of total RNA used in the reverse transcriptase reaction. A general decrease in the mRNA level of the six genes was seen during the first 18 hrs (Figure 7), probably as many leucocytes were going into apoptosis. Apoptosis is a tightly regulated process involving activation and inhibition of several genes at the transcriptional level and prelytic DNA fragmentation [50,51]. The up-regulation of such apoptosis-regulated genes can lead to difficulties in the analysis of real time RT-PCR data, as the stability of the reference genes may be affected by the apoptotic cells. Also, during the first 18 hrs post-stimulation the mRNA levels of the six reference genes were generally lower in the anterior kidney leucocytes stimulated with ConA-PMA compared to the non-stimulated control cells. Various apoptosis-related genes and immune-response genes were isolated in a cDNA library based on anterior kidney leucocytes stimulated with ConA-PMA from Japanese flounder [52,53]. This indicates an up-regulation of such genes in response to ConA-PMA stimuli, and could be diluting or regulate the expression of reference gene mRNA. Interestingly, at 24 hrs the expression of ACTB1, HPRT1, and Tubb2C compared to the levels at 18 hrs were increased in the non-stimulated cells that were isolated from *in vivo *NNV stimulated fish, and in the cells stimulated *in vitro *with ConA-PMA. However, due to high variation, the small sample size (n = 4), and as the experimental design was limited regarding the effect of tanks, no conclusion could be drawn.

When analyzing the data from anterior kidney leucocytes by NormFinder, geNorm, and BestKeeper, the software programs deviated in the ranking of the six genes (Table 4). UbcE and RPL7 were generally listed as good candidates, followed by EF1A1 and HPRT1, and with ACTB1 and Tubb2C at the bottom having the lowest rankings. Despite the good ranking of UbcE and RPL7, the difference in reference gene expression through time and between the groups renders them not optimal for normalization of real time RT-PCR data from ConA-PMA stimulated anterior kidney leucocytes. This is in contrast to the findings in Atlantic salmon where EF1A and ACTB1 were found suitable as reference genes in LPS stimulated anterior kidney leucocytes 72 hrs post stimulation [47]. However, it should be noted that in this study only one time point was sampled, thus the variation seen over time in this study cannot be directly compared. In three-spined stickleback (*Gasterosteus aculeatus*), hardly any of the 9 genes tested in anterior kidney cell cultures, including ACTB1, EF1A1, and HPRT1, were expressed with SD lower than 1 [54]. UbcE having a SD of 0.5 in non-stimulated control cells (Table 3) could be a good candidate for normalization of anterior kidney leucocytes in other experimental setups not using ConA-PMA as stimuli.

## Conclusion

This study reports the expression level of six commonly used reference genes during halibut development, in different tissues of healthy and NNV-infected halibut, and in anterior kidney leucocytes both *in vivo *and *in vitro *stimulated. The study emphasizes the need for such pilot studies, as universal reference genes have not been identified. Generally, it was found that EF1A1 and RPL7 were the genes that showed the least variation, with HPRT1 and UbcE as intermediate genes, and ACTB1 and Tubb2C as the least stable ones. However, EF1A1 and RPL7 cannot be recommended for a two-gene normalization strategy, as both genes are involved in protein biosynthesis and some co-regulation may be expected. During development, UbcE was found to be the most stable reference gene during activation of zygotic transcription. But, some stage specific expression is seen, and it can be questioned if the use of maternal and zygotic transcripts as references within the same experiment is optimal. EF1A1 and RPL7 were found to be the best candidate reference genes for a normalization approach in halibut larvae and juveniles, and in tissue samples from one year old halibut. This is the first report exploring reference gene expression during a NNV infection in halibut, and amongst the genes tested especially RPL7 was shown to be a good candidate, but also EF1A1 and HPRT1. Based on this data set, none of the six housekeeping genes were optimal as reference gene candidates in ConA-PMA stimulated leucocytes, but UbcE could be a good candidate in other experimental setups showing relatively stable expression in the non-stimulated control groups. This study will facilitate further work on developmental processes and will help in studying immune responses during host-pathogen interactions in Atlantic halibut, both *in vivo *and *in vitro*.

## Authors' contributions

ACØ was involved in the design and sampling of adult fish and NNV-infection experiment, responsible for the sequencing, real time RT-PCR, data analysis, and drafted the manuscript. AHN participated as a supervisor in the study design and analysis, and helped in drafting the manuscript. SP was involved in study design and sampling in all the studies, and helped in drafting the manuscript. All authors read and approved the final manuscript.
